# The lockdown effect: A counterfactual for Sweden

**DOI:** 10.1371/journal.pone.0249732

**Published:** 2021-04-08

**Authors:** Benjamin Born, Alexander M. Dietrich, Gernot J. Müller

**Affiliations:** 1 Frankfurt School of Finance & Management, CEPR, and CESifo, Frankfurt, Germany; 2 University of Tübingen, Tübingen, Germany; 3 University of Tübingen, CEPR, and CESifo, Tübingen, Germany; Health Directorate, LUXEMBOURG

## Abstract

While most countries imposed a lockdown in response to the first wave of COVID-19 infections, Sweden did not. To quantify the lockdown effect, we approximate a counterfactual lockdown scenario for Sweden through the outcome in a synthetic control unit. We find, first, that a 9-week lockdown in the first half of 2020 would have reduced infections and deaths by about 75% and 38%, respectively. Second, the lockdown effect starts to materialize with a delay of 3–4 weeks only. Third, the actual adjustment of mobility patterns in Sweden suggests there has been substantial voluntary social restraint, although the adjustment was less strong than under the lockdown scenario. Lastly, we find that a lockdown would not have caused much additional output loss.

## Introduction

As the COVID-19 pandemic spreads across the globe, policy makers are faced with a trade-off. By imposing a lockdown, they may limit the spread of COVID-19 infections and deaths. But a lockdown might also entail severe economic and social costs [1, 2]. In order to inform policy makers, it is essential to quantify both the costs and the benefits of lockdowns. In this study, we focus on the health benefits and quantify the extent to which a lockdown limits the spread of COVID-19. We only make a first pass at quantifying the economic costs and ignore the social costs altogether.

A major challenge in quantifying the lockdown effect is that infection dynamics are bound to change over time even in the absence of a lockdown—at least for two reasons. First, the basic Susceptible-Infectious-Recovered (SIR) model assumes a constant infection probability, but predicts that infection growth slows over time as the pool of infected and susceptible people shrinks [3]. Second, people may adjust their behavior in the face of infection risk and restrain their social interactions voluntarily. This, too, impacts infection growth. Several studies neglect these aspects and attribute any change in the growth rate of infections to lockdown measures or, more broadly speaking, to non-pharmaceutical interventions (NPI). NPI are thus found to be very effective in reducing infection growth [4] and to reduce the number of deaths by about 95% according to an influential study [5].

In the present study, we explicitly account for changing infection dynamics as we quantify the lockdown effect on the basis of a purely empirical approach. Specifically, we focus on Sweden: it stands out from its European peers in that its government opted against a lockdown in the first half of 2020, although its exposure to COVID-19 was not systematically different from the rest of Europe. Still, the Swedish authorities merely advised—rather than ordered—citizens to adjust their behavior in the face of the pandemic. For instance, people were told “to avoid unnecessary traveling and social events, to keep distance to others, and to stay at home” if they had any symptoms. In addition, those above age 70 were “advised to avoid social contact” and “visits to retirement homes” were banned [6].

In order to quantify the lockdown effect, we benchmark actual developments in Sweden against a counterfactual lockdown scenario approximated through the outcomes in a synthetic control unit [7]. We construct it on the basis of a donor pool of European countries that actually imposed a lockdown and by making sure that it resembles Sweden in the way the pandemic unfolded *before* the lockdown. In the counterfactual scenario, Sweden would have imposed a first lockdown, just like in the control unit, running for 9 weeks from March 15 to May 17, 2020.

We find that it takes 3 to 4 weeks for the lockdown to make a difference. Only after this period do we observe fewer COVID-19 infections and deaths in the control unit. The cumulative difference grows over time, but only up to the end of August. For this reason, we limit our assessment of the lockdown effect to the period up to September 1, 2020. We consider alternative specifications to construct the control unit based on COVID-19 infections and deaths and, based on these two baseline specifications, find that a lockdown would have reduced infections and deaths, in turn, by 75% and 38%, respectively. We obtain similar numbers for various robustness tests, just like a recent study that follows our empirical strategy closely [8]. Our results are also in the same order of magnitude as those reported in a case study of Norway and Denmark, which uses Sweden as a control group [9].

We also analyze Google mobility reports and find a profound change of actual mobility patterns in Sweden, even as no lockdown is imposed: people travel less and spend more time at home or in parks. The extent of this adjustment is sizable, but not as strong as in the control unit under the lockdown. This finding suggests that voluntary social restraint limits infection growth to a considerable extent—in line with recent work that augments the basic SIR model to account for behavioral adjustments in the face of infection risk [10–12]—and may rationalize why the lockdown effect becomes manifest with a considerable delay only. Another explanation for the delay is that in case of mandatory measures compliance may not be taken for granted [13, 14].

Yet overall our results also support the notion that there is a non-trivial infection externality: while people adjust their behavior to limit their own exposure to others, the adjustment falls short of what is socially optimal because they do not account for the infection risk to which they expose others once they become infected. Lastly, we find that, against the backdrop of the large actual output loss in the first half of 2020, a lockdown would have caused little additional output loss in Sweden.

## Materials and methods

Our empirical strategy is centered around the notion that one may approximate a counterfactual lockdown scenario for Sweden by the outcomes in a synthetic control unit. We construct the *control unit* on the basis of a *donor pool* of countries in such a way that it behaves just like Sweden *before* the lockdown. In contrast to Sweden, all countries in the donor pool imposed a lockdown in response to the first wave of COVID-19 infections during the first half of 2020. Any difference in infection dynamics or COVID-19 related deaths in Sweden and the control unit *since* the lockdown may then be attributed to the lockdown in the control unit. In what follows we first provide details on the donor pool before turning to the construction of the control unit. As supporting information we also show the time series for infections and deaths in each country (S1 Fig in S1 File).

### The donor pool

We construct the control unit below as a weighted average of the countries in the donor pool. To ensure a high degree of homogeneity between Sweden and the control unit, we restrict the donor pool to Norway and western EU countries with more than 1 million inhabitants. In total, it includes 13 countries. Table 1 provides details on each country in the donor pool, including the timing of the lockdown and the most important measures. The first lockdown was imposed in Italy on March 9, the last in the Netherlands on March 24. These lockdowns typically involved the closing of non-essential shops as well as a ban on gatherings of more than two people. In some instances, the ban applied only to gatherings of 10 people or more. In Sweden only gatherings of more than 50 people were banned.

**Table 1:** Donor pool and Sweden during the first wave: Lockdown measures and dates.

Country	Lockdown		Containment Measures	Day 1	Days to Lockdown	Days in Lockdown	Lockdown Stringency	% of control unit
	Start	End						
Austria	16.03.	01.05.	non-essential shops closed, ban on gatherings of more than 5 people	29.02.	16	46	83	00.0
Belgium	18.03.	11.05.	non-essential shops closed, ban on gatherings of more than 2 people, stay-at-home order	03.03.	15	54	81	00.0
Denmark	18.03.	11.05.	non-essential shops closed, ban on gatherings of more than 50 people	03.03.	15	54	70	30.0
Finland	16.03.	01.06.	government agencies closed, ban on gatherings of more than 10 people, stay-at-home advice	01.03.	15	77	56	25.3
France	17.03.	11.05.	non-essential shops closed, ban on gatherings of more than 2 people, stay-at-home order	29.02.	17	55	90	00.0
Germany	23.03.	06.05.	non-essential shops closed, ban on gatherings of more than 2 people	01.03.	22	44	73	0.00
Greece	23.03.	11.05.	non-essential shops closed, ban on gatherings of more than 10 people, stay-at-home-order	05.03.	18	46	82	00.0
Ireland	28.03.	08.06.	non-essential shops closed, stay-at-home-order	04.03.	24	72	87	00.0
Italy	09.03.	18.05.	non-essential shops closed, stay-at-home-order	22.02.	16	70	85	00.0
Netherlands	24.03.	11.05.	non-essential shops closed, ban on gatherings	02.03.	22	48	79	25.8
Norway	13.03.	01.06.	restaurants, bars closed, ban on gatherings of more than 5 people (24.03)	28.02.	14	80	66	15.0
Portugal	19.03.	01.06.	no shops closed, government agencies closed, stay-at-home advice	06.03.	13	74	79	00.0
Spain	14.03.	08.06.	non-essential shops closed, stay-at-home-order	01.03.	13	86	78	03.9
Sweden	-	-	No Lockdown imposed, ban on gatherings of more than 50 people	28.02.	-	-	42	-

Notes: Donor pool includes western EU countries with population size of at least one million and Norway. Day 1 is the day when the number of infections surpasses a threshold of one infection per one million inhabitants. Sources for lockdown dates and details are provided as supporting information (S1 Table in S1 File). Lockdown ends when shops were reopened, except for Finland and Norway (restaurant reopening). Lockdown stringency is average value during lockdown period based on data from the Coronavirus Government Response Tracker [15]. For Sweden, average is for counterfactual lockdown period from March 15 to May 17, 2020 (Specification A). “% of control unit” pertains to baseline (Specification A), rounded to first digit.

As a comprehensive measure, we also report the average stringency of the lockdown based on data from the Coronavirus Government Response Tracker [15]. As can be seen in the second-to-last column of Table 1, there is some variation in lockdown stringency across countries. According to this measure, there has been some response of the Swedish authorities to the Coronavirus, but—consistent with the premise of our analysis—the index number is considerably lower than for the other countries in the donor pool.

In order to assess the impact of the lockdown, it is essential to ensure that the development of the pandemic is comparable across countries prior to the lockdown. And because the virus arrived at different dates in each country, we may not compare countries on a calendar-day basis. Therefore, we initialize observations for each country using a common reference point: for our baseline we define day 1 as the day when the number of infections surpasses a threshold of one infection per one million inhabitants. Day 1 varies from country to country, see Table 1. For instance, in Sweden it is February 28, in Norway it happens to be the same day, while in Denmark it is March 3. Given day 1, we find that it takes countries at least 13 days since day 1 to impose a lockdown. Its duration also varies across countries from a minimum of 44 days in Germany to a maximum of 86 days in Spain, see again Table 1. In our robustness analysis, we consider alternative definitions for day 1 and find that our results are similar to what we obtain for the baseline.

### Constructing the control unit

For our baseline, we construct the control unit as follows. We require it to track the infection dynamics in Sweden during the first 13 days as closely as possible, that is, before any country in the donor pool imposed a lockdown. This is a long time period in the context of our analysis because COVID-19 infections spread rapidly and our identification assumption is that infection dynamics during this 13-day window are informative about a country’s overall exposure to the virus. Moreover, since the number of infections are initially very low, we target the log of infections rather than the level. In this way, we make sure that the early observations within the 13-day window play a non-negligible role for the construction of the control unit.

In addition, the control unit should be comparable to Sweden in terms of population density and size and, in particular, in its age distribution because these factors may play an important role for infection dynamics. For this reason, we target three additional observations: population size, the share of the population older than 65 years, and the share of the urban population. In total, we target 16 observations in order to construct the control unit: log infections at daily frequency within the 13 day window prior to the first lockdown and the three covariates. In a robustness test, we also include the number of tests prior to the lockdown in the set of covariates and find that this does not alter our results.

Formally, we select weights on the countries in the donor pool for which we obtain the best match between the control unit and Sweden for the 16 target observations. That is, we let **x**_**1**_ denote the (16 × 1) vector of observations in Sweden and let **X**_**0**_ denote a (16 × 13) matrix with observations in the countries included in the donor pool. Finally, we let **w** denote a (13 × 1) vector of country weights *w*_*j*_, *j* = 1, …, 13. Then, the control unit is defined by w* which minimizes the following mean squared error:

$$\begin{array}{c} {\left(x_{1}-X_{0}w\right)}^{\prime }V\left(x_{1}-X_{0}w\right), \end{array}$$ (1)

subject to *w*_*j*_ >= 0 for *j* = 1, …, 13 and $\sum_{j=1}^{13}w_{j}=1$. In this expression, **V** is a (16 × 16) symmetric and positive semidefinite matrix. Here, **V** is a weighting matrix assigning different relevance to the characteristics in **x**_**1**_ and **X**_**0**_. Although the matching approach is valid for any choice of **V**, it affects the weighted mean squared error of the estimator [7]. We choose a diagonal **V** matrix such that the mean squared prediction error of the outcome variable (and the covariates) is minimized for the pre-treatment period [7, 16].

A widely discussed shortcoming of the available infection data is that the number of detected cases is not independent of the number of tests, since infections may go undetected if symptoms are mild or even absent. Hence, we verify—once we have constructed the control unit—that the frequency of testing in Sweden does not change systematically relative to the control unit in our sample period. Importantly, while we observe some variation in the relative testing frequency, there is no systematic variation that would account for our results. We show the time series of relative testing as (S2 Fig in S1 File).

Still, in order to verify that the results based on matching infection data (Specification A) are not distorted by measurement problems, we consider an alternative strategy to construct the control unit (Specification B): we match the number of deaths rather than the number of infections. Since initially there are no or only very few deaths, we need to specify day 1 differently in this case. We define it as the day when, in a given country, the number of deaths surpasses one per 100.000 inhabitants. Otherwise we proceed in the same way as in the baseline (Specification A). Notably, we match the same set of covariates and target the log of the number of deaths for 13 days after day 1. As a result, the matching period includes observations from the lockdown period in several instances. This is unlikely to distort our results, however, because the time lag between COVID-19 cases and deaths is estimated to be 4-6 weeks [17].

## Results

In what follows, we present three sets of results. First, we quantify the lockdown effect, measured as the difference in outcomes in Sweden and the control unit, both for Specification A and B. Second, we present results regarding the extent of voluntary social restraint in Sweden and the control unit. Lastly, we make a first pass at assessing the potential economic costs of a lockdown in Sweden.

Our results for the baseline Specification A are based on a control unit which is composed of five countries: Denmark (30.0%), Finland (25.3%), the Netherlands (25.8%), Norway (15.0%), and Spain (3.9%). The other countries in the donor pool receive negligible weights only, as reported in the right column of Table 1. Note that the number of countries with non-negligible weight is not restricted by our procedure and may vary across specifications. Using these weights, we compute the average outcome across the countries in the control unit in order to approximate the counterfactual infection outcome for Sweden. On average, it takes 16 days after day 1 before a lockdown is imposed. Its stringency is 68 and hence well above the actual value for Sweden. Since day 1 in Sweden is February 28, 2020, we assume that a counterfactual lockdown in response to the first wave of COVID-19 infections would have run from March 15 to May 17, 2020, that is, for 9 weeks.

The weights for Specification B are obtained by matching the number of deaths rather than the number of infections. In this case, the control unit is composed of Belgium (57.5%), Finland (17.4%), Italy (0.1%) and Portugal (25.0%) and the lockdown period runs from March 14 to May 16. Before measuring the lockdown effect, we verify that the construction of the control unit is not driven by the time dependency of the pandemic in different countries. For this purpose we correlate the weight of a country in the control unit with the difference of day 1 in that country and day 1 in Sweden. Reassuringly, we find the correlation is basically zero for Specification A and Specification B, see S3 Fig in the S1 File.

### The lockdown effect

**Fig 1:**
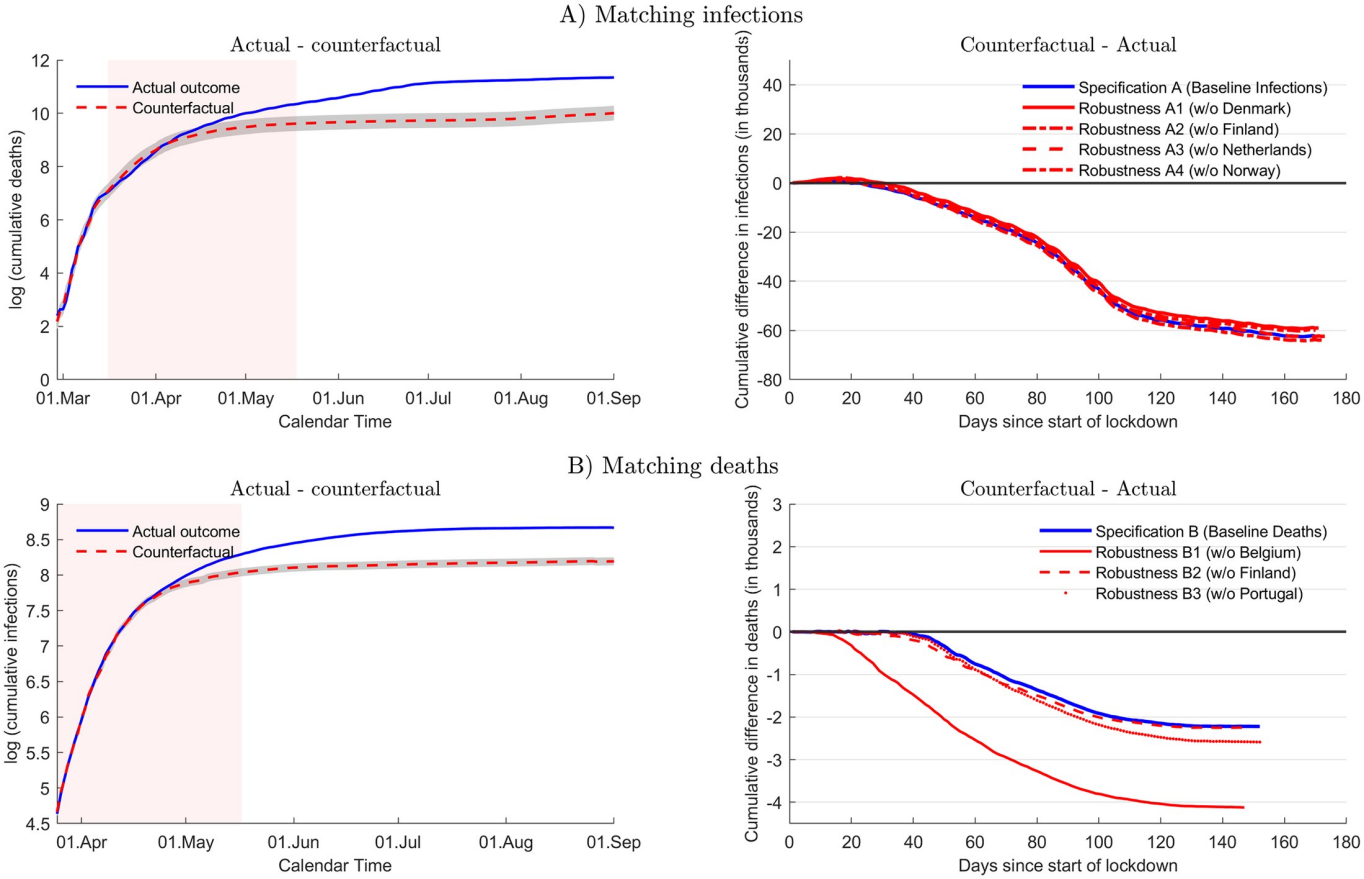
COVID-19 infections in Sweden. Top-left panel compares actual infections (blue solid line) and control unit/counterfactual (red dashed line) in logs for baseline Specification A. Top-right panel shows difference of infections between counterfactual and actual outcome in thousands. Table 1 reports country weights for control unit counterfactual (Specification A (baseline)). Gray shaded area shows two standard deviations of difference between infections in Sweden and control unit during the first 13 days (matching period). Pink shaded area: lockdown period (March 15–May 17). Specifications (A1–A4): alternative control units based on restricted donor pools. Bottom panels organized in the same way, but for Specification B.

We contrast the actual outcome in Sweden and in the control unit in Fig 1. The top panels are based on the baseline (Specification A), the bottom panels are based on Specification B. The top-left panel of Fig 1 shows infection dynamics in Sweden (blue solid line) and the control unit that approximates the counterfactual outcome (red dashed line). The vertical axis measures cumulative infections in logs, the horizontal axis represents calendar days. The lockdown period is indicated by the pink shaded area. By construction, the control unit tracks infection dynamics closely before the lockdown. We also obtain a good fit for the fraction of the population aged 65 or above (0.201 in Sweden and 0.197 in the control unit, respectively), population size (10.175 millions in Sweden and 10.186 in the control unit) and for the urbanization rate (0.874 and 0.871, respectively). The gray shaded area represents two standard deviations of the difference between log infections in Sweden and the control unit during the matching period, an informal measure to put deviations since the start of the lockdown into perspective [18]. We observe that infection dynamics start to diverge visibly midway through the lockdown period: at this point infection growth slows markedly in the control unit, that is, in the counterfactual lockdown scenario relative to the actual developments in Sweden.

The top-right panel of Fig 1 displays the difference in infections between the control unit (counterfactual) and Sweden, measured as the cumulative difference in terms of the number of new infections since the beginning of the lockdown in the counterfactual. Initially, the difference is small, but over time, infection growth in Sweden is higher, such that the difference turns negative. The blue solid line represents our baseline (specification A) for which we report country weights in Table 1.

The same panel shows results for alternative specifications based on a restricted donor pool: in each case, we exclude, in turn, each country that receives a sizeable weight in the baseline specification. In this way we verify that our results are not driven by any specific country in the donor pool. In each instance, we obtain a new control unit that differs from the baseline in terms of composition and also somewhat in the timing and the stringency of the lockdown, as detailed in the (S2 Table and S4-S12 Figs in S1 File). Yet, as the right panel of Fig 1 shows, a robust picture emerges across specifications: a sizeable lock-down effect materializes with a delay of 20-30 days.

The bottom panels of Fig 1 are organized in the same way, but pertain to Specification B, that is, they show results that we obtain as we match the number of deaths rather than infections. In the bottom-left panel, we observe that towards the end of the lockdown period the number of deaths are distinctly lower in the control unit/counterfactual. In the lower-right panel we display the difference between the control unit and the actual outcome in terms of deaths for Specification B as well as for Specifications B1 to B3. These specifications are based on restricted donor pools: we remove again those countries from the donor pool which receive a large weight in Specification B.

Fig 1 also shows that the effect of the lockdown is not limited to the lockdown period. The actual developments continue to diverge from the counterfactual developments, both according to Specification A and B. However, the effect of the lockdown levels off at some point. In order to measure its overall effect, we use September 1 as a cutoff date. Specifically, Table 2 reports the total number of new infections and deaths between the start of the lockdown (which differs slightly across specifications because of the changing composition of the control unit), contrasting once more the developments in Sweden and the control unit (counterfactual). The top panel shows numbers for the baseline Specification A: 83,375 new infections in Sweden vs 20,738 in the counterfactual lockdown scenario. This is our main result: a lockdown would have reduced the number of infections in Sweden by 75%. We use the weights obtained for Specification A to compute the counterfactual outcome for deaths. Table 2 also reports results for the alternative specifications considered in Fig 1 above: we find similar values than for the baseline, both for infections and for deaths.

**Table 2:** The lockdown effect on COVID-19 infections and deaths.

		Infections		Deaths	
		Actual	Counterfactual	Actual	Counterfactual
**A)**	**Baseline (Matching Infections)**	**83,458**	**21,150**	**5,806**	**3,013**
			**-75%**		**-48%**
A1)	W/o Denmark in donor pool	83,458	24,450	5,806	2,523
			-71%		-57%
A2)	W/o Finland in donor pool	83,527	19,606	5,808	1,065
			-77%		-82%
A3)	W/o Netherlands in donor pool	83,598	21,209	5,810	4,302
			-75%		-26%
A4)	W/o Norway in donor pool	83,375	23,520	5,805	2,589
			-72%		-55%
**B)**	**Baseline (Matching Deaths)**	**79,122**	**57,655**	**5,795**	**3,573**
			**-27%**		**-38%**
B1)	W/o Belgium in donor pool	76,080	35,307	5,777	1,653
			-53%		-71%
B2)	W/o Finland in donor pool	77,207	56,576	5,790	3,541
			-27%		-39%
B3)	W/o Portugal in donor pool	78,505	51,428	5,795	3,207
			-34%		-45%

Notes: New infections and deaths since start of lockdown up until September 1, 2020. Figures for the actual outcome differ across specifications because the start of the lockdown period varies with the composition of the control unit. Panel A: baseline matching infections (see Fig 1). Panel B: baseline matching deaths (see Fig 1). Panels A1-A4 and B1-B3: alternative control units, for which each of the countries with non-negligible weight in baseline control unit, in turn, is excluded from donor pool. See text for details.

In the lower panel of Table 2 we repeat the exercise for Specification B. In this case, we directly target the number of deaths and find in particular that a lockdown would have reduced the number of deaths by 38%, from 5,795 to 3,573. This also serves as a cross-check: by and large, the results are similar to what we obtained under Specification A. Across specifications the lockdown effect for infections ranges between 27 and 77 percent, and between 26 and 82 percent for deaths. We also assess whether our results are sensitive to the specific definition of day 1. S3 Table in the S1 File reports results: they turn out to be similar to what we obtain for the baseline.

Finally, we verify that the lockdown effect is indeed caused by a counterfactual lockdown, or put differently, by the lockdown that takes place in the control unit but not in Sweden, and is not purely random. To assess this formally, we run a number of placebo tests [7, 19]. In doing so, we base our assessment on the relative root mean square error [18]: we replace Sweden with, in turn, each of the countries in the donor pool and construct a control unit in each instance—in the same way as we do for Sweden, both for Specification A and B. Next we compare the deviation of the actual outcome in each country from its control unit and summarize it by the root mean square error (RMSE), both before and after the matching period. Last, we compare the ratio of the RMSE after the matching period to the RMSE from before the matching period. In this way, we normalize the deviation after the matching period by the deviation during the matching period. S4 Table in the S1 File provides additional details and reports results: the relative RMSE is by far the largest in the case of Sweden. This suggests that the lockdown (or rather the absence thereof) is indeed causing the difference between the actual developments in Sweden and the control unit.

### Social distancing

Our results show that a lockdown would have reduced the number of COVID-19 infections and deaths in Sweden, but this lockdown effect starts to materialize with a delay of 3 to 4 weeks only. In order to rationalize this result, we compare the extent of social distancing in Sweden and in the control unit on the basis of Google COVID-19 Community Mobility Reports [20]. The reports classify locations on the basis of six distinct categories, namely, Work, Transit, Retail and Recreation, Grocery and Pharmacy, Parks, and Residential and measure the change in the number and the length of stays at these locations relative to the median value of the same weekday between January 3 and February 6, 2020. To construct the control unit, we use the country weights obtained for Specification A above.

**Fig 2:**
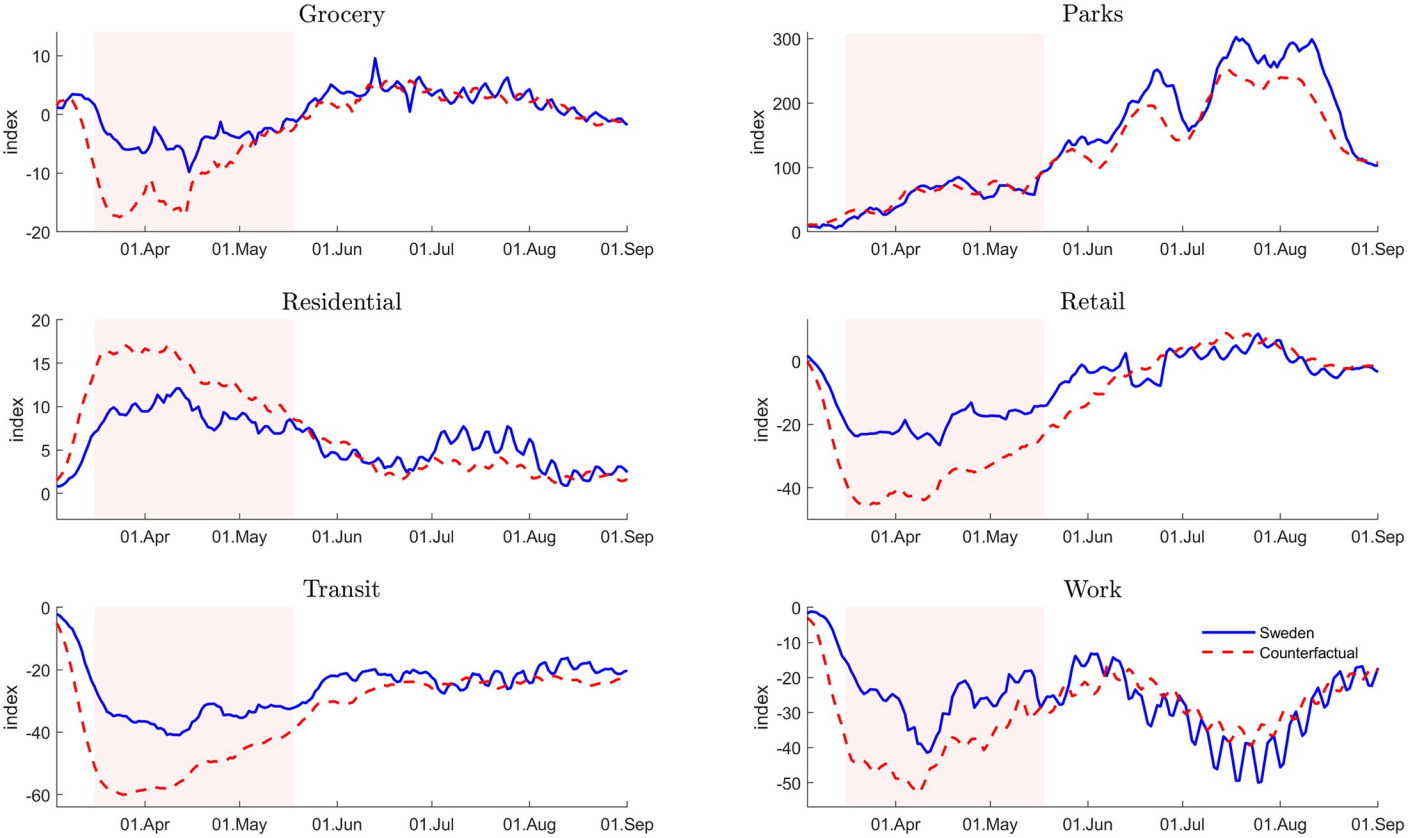
Mobility adjustment in Sweden. Actual outcome (blue solid line) vs control unit/counterfactual (red dashed line). Vertical axis measures percentage change relative to median in early 2020. Horizontal axis measures 11-day symmetric moving average. Pink shaded area indicates lockdown period. Construction of counterfactual: see Fig 1 (Specification A, Baseline). Data source: Google mobility reports [20].

Fig 2 displays mobility adjustments for each location, contrasting once more actual data for Sweden (blue solid line) and for the control unit (red dashed line) that is meant to approximate the counterfactual outcome in case of a lockdown. We measure observations using 11-day symmetric averages along the horizontal axis, and the percentage change relative to the pre-COVID-19 period along the vertical axis. As before, the pink shaded area indicates the lockdown period. Several findings stand out. First, we observe a pronounced decline of mobility in the top four panels. They provide a measure of activities associated with travel and work as well as shopping and dining in restaurants (“recreation”). At the same time, people spend more time in parks and at home (bottom panels). More importantly still, the adjustment starts to take place before the lockdown period and can be observed both in Sweden and in the control unit. Consistent with these findings, it has been documented elsewhere that COVID-19 has induced job seekers to reduce their search intensity and employers to reduce vacancy postings in the absence or prior to a lockdown [21, 22]. Last, we observe that while the adjustment of activities follows roughly the same pattern, it is more pronounced for the control unit during the lockdown period.

### The economic costs of a lockdown

Based on our approach, we also compute the economic costs of the lockdown in terms of forgone output growth. For this purpose we compare GDP growth in Sweden in the first two quarters of 2020 to GDP growth in the control unit. To ensure comparability, we normalize GDP growth by subtracting the average growth rate in 2019 in both instances. For the first and second quarter of 2020, we find that on a year-on-year basis GDP growth in Sweden has been 0.61 and 9.03 percentage points lower compared to 2019. In the control unit, the numbers are 2.25 and 9.74, respectively. Hence, here too we find a lockdown effect: a lockdown in Sweden would have further reduced annualized output growth by 1.64 and 0.71 percentage points, in the first and second quarter of 2020, respectively.

## Discussion

Our analysis shows that while a lockdown may limit the spread of COVID-19 infections and reduce the number of deaths, it takes time for this effect to materialize. This is noteworthy because the incubation period for COVID-19 is on average only 5-6 days, and at most up to 14 days [23]. The delay with which a lockdown effect becomes visible may be due to the time span between infection and testing; yet it may also take time for the lockdown to make a difference because of voluntary social distancing which takes place in the absence of a lockdown.

And indeed we find that people adjust their behavior in response to pandemic whether there is a lockdown or not: they reduce their social interactions in ways which limit the spread of the virus—there is, in other words, voluntary social restraint. This point emerges also clearly from a number of recent model-based contributions (referenced in the introduction above) that have extended the basic SIR model to account for key insights from economic theory, namely by allowing for peoples’ (rational) adjustment of work, consumption and leisure activities in the face of infection risk. More generally, the idea is to model explicitly the exposure to the virus (of those people which are susceptible), as in the Susceptible-Exposed-Infectious-Recovered (SEIR) model which has been analyzed extensively in the context of the COVID-19 pandemic [24–26]. A systematic review of this body of work is beyond the scope of this paper. We highlight, however, what is an important result in the context of our empirical analysis: model simulations illustrate that voluntary social restraint can have large effects, both in terms of limiting the maximum share of infections in the population and in terms of inducing an economic contraction [10].

That said, economic theory also identifies an infection externality: while people adjust their behavior to limit their own exposure to the virus, the adjustment is weaker compared to what would be socially optimal because they do not account for the infection risk which they represent for others. This provides a rationale for government-imposed NPI and an explanation why the outcomes under “laissez faire” and the optimal policy can be quite different. In Sweden it takes a considerable amount of time for the difference to become visible—likely because lockdown policies in the control unit have been in all likelihood less well targeted than the optimal policy in model-based simulations. Still, our results show that, over time there is a sizeable lockdown effect—in line with the notion of a non-trivial infection externality [27].

In addition, our analysis suggests that the economic costs of a lockdown in Sweden would have been moderate, consistent with the finding that the Swedish labor market performed only slightly better than that of its neighbors [28]. This, too, may be partly explained by voluntary social distancing because it has taken a sizeable toll on output growth in Sweden. In fact, mobility is a good proxy for how social distancing—voluntary or mandatory—affects economic activity [29]. In the case of Sweden, a lockdown may simply not have raised the economic costs much further.

However, the economic costs of a lockdown in any given country also depend on what happens in the rest of the world. If, as in the case of Sweden, all major trading partners are put under lockdown, economic growth declines even as no lockdown is imposed. Against this background it would be interesting to investigate an alternative counterfactual in which no country imposes a lockdown. Such an assessment, however, is beyond the scope of the present study.

Before concluding, we should point out a number of further limitations of our analysis. First, we use data on COVID-19 infections and deaths even though there are serious issues related to measurement, not least the fact that the number of reported infections depends on the number of tests. Still, our analysis is based on the same data that informs public discussions and actual policy design.

Second, we assume a macro perspective throughout and study the effect of “a” lockdown (with stringency 68). Clearly, specific lockdown measures may differ strongly in terms of effectiveness. For instance, it has been shown on the basis of an approach similar to ours that making face masks mandatory is fairly effective in limiting the spread of infections [30].

Third, there is the issue of external validity: we have developed a counterfactual for Sweden and cannot be sure that results carry over to other contexts and countries. In particular, we cannot rule out that the behavioral adjustment in Sweden was influenced by the fact that other countries in Europe imposed a lockdown. We note, however, that our results are similar to those obtained for California, the first state in the US to issue a shelter-in-place order, on the basis of an approach similar to ours [31].

Last, we stress that our study focuses on benefits of a lockdown in terms of limiting COVID-19 infections and deaths. We find that these benefits are not trivial. While we also quantify the costs of a lockdown in terms of output and find the effect to be rather moderate, our analysis is altogether silent on the social, political, and psychological costs of a lockdown. Hence, the final verdict on lockdowns as a policy tool is still out.

## Conclusion

Our study focuses on the Swedish case to quantify the effect of a lockdown on COVID-19 infections and deaths. We may summarize our results in four findings: First, we find the effect to be sizable and robust across specifications: it ranges from -27% to -77% for infections and from -26% to -82% for deaths. Second, the lockdown effect starts to materialize with a delay of 3–4 weeks only. Third, the actual adjustment of mobility patterns in Sweden suggests there has been substantial voluntary social restraint, although the adjustment was less strong than under the lockdown scenario. Lastly, we find that a lockdown would not have caused much additional output loss.

## Supporting information

S1 File (PDF)
