# Supplementary material for: The lockdown effect: A counterfactual for Sweden
Source: PLoS One. 2021 Apr 8;16(4):e0249732. doi: 10.1371/journal.pone.0249732 (PMC8031244; doi:10.1371/journal.pone.0249732)
Supplement: S1 File — (PDF) [file pone.0249732.s001.pdf]

# S1 File

# The lockdown effect: A counterfactual for Sweden

**Benjamin Born, Alexander M. Dietrich, Gernot J. Müller**[1]

**S1. Data sources.** Our data source for the number of COVID-19 infections and deaths up to September 1, 2020 is the Johns Hopkins University Dong et al., 2020. Observations are available at daily frequency. They are assembled using national (e.g. ministry of health, government) as well as international official sources (e.g. WHO, European Centre for Disease Prevention and Control). We display the time series data for each country in the donor pool in Figure A.1. Finally, our data for the population size, the fraction of age 65 and above, and the urbanization rate is provided by the World Bank Worldbank, 2019 Revision(a),(b),(c).

We use Google COVID-19 Community Mobility Reports to measure mobility changes due to the pandemic Google, 2020. They are available for each country in our donor pool and provide a measure for how long and how frequently certain types of locations are visited. Google collects location data in various ways using mobile phone positions (via mobile networks or GPS data), a user's IP address, search queries, or navigation requests. Google uses this information only if users actively agree to share their "Location History". Last, in order to compare GDP growth in Sweden and the control unit we use OECD data OECD, 2020.

To control for the frequency of testing in Sweden and the donor pool countries, we rely on data from the European Center for Disease Prevention and Control European Centre for Disease Prevention and Control, 2020.

[1]Born: Frankfurt School of Finance & Management, CEPR, and CESifo, b.born@fs.de, Dietrich: University of Tübingen, alexander.dietrich@uni-tuebingen.de. Müller: University of Tübingen, CEPR, and CESifo, gernot.mueller@uni-tuebingen.de.

**S1 Table:** Lockdown Sources.

| Country | Lockdown Start Source | Lockdown End Source |
|---|---|---|
| Austria | *www.parlament.gv.at* | *www.wko.at* |
| Belgium | *www.belgium.be* | *www.belgium.be/en* |
| Denmark | *politi.dk* | *politi.dk* |
| Finland | *valtioneuvosto.fi* | *valtioneuvosto.fi* |
| France | *www.diplomatie.gouv.fr* | *www.tagesschau.de* |
| Germany | *www.bundesregierung.de* | *www.bundesregierung.de* |
| Greece | *gr.usembassy.gov/covid-19* | *www.visitgreece.gr* |
| Ireland | *www.gov.ie* | *www.gov.ie* |
| Italy | *www.trovanorme.salute.gov.it* | *www.salute.gov.it* |
| Netherlands | *www.government.nl* | *www.government.nl* |
| Norway | *www.helsedirektoratet.no* | *www.regjeringen.no* |
| Portugal | *www.acm.gov.pt* | *www.acm.gov.pt* |
| Spain | *www.gov.uk/foreign-travel-advice/spain/coronavirus* | *www.bbc.com* |

**S2. Time series Data for Countries in Donor Pool: Infections and Deaths.** Figure A.1 presents the raw data series for deaths and infections of the countries in the donor pool, as made available by the Johns Hopkins University Dong et al., 2020. Data displayed runs from February 22 to September 1.

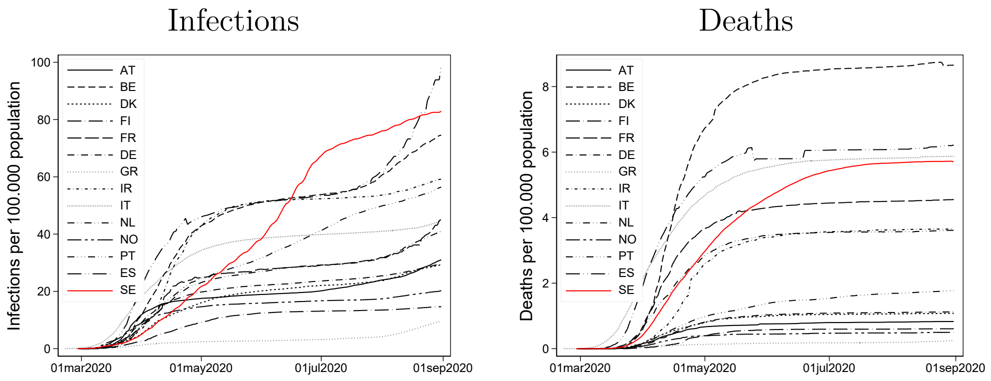


S1 Fig: Infections and Deaths. Left panel shows cumulative infections per 100.000 population in countries of donor pool. Right panel shows cumulative COVID-19 deaths per 100.000 population in countries of donor pool. Red line represents data for Sweden. Data source: Dong et al., 2020.

**S3. Ratio of Tests.** In Figure A.2, we display the ratio of weekly tests between Sweden and the control unit. We use weekly data provided by European Centre for Disease Prevention and Control, 2020. Data for each country is then shifted in time so that the countries' day 1 is in the same week as for Sweden.

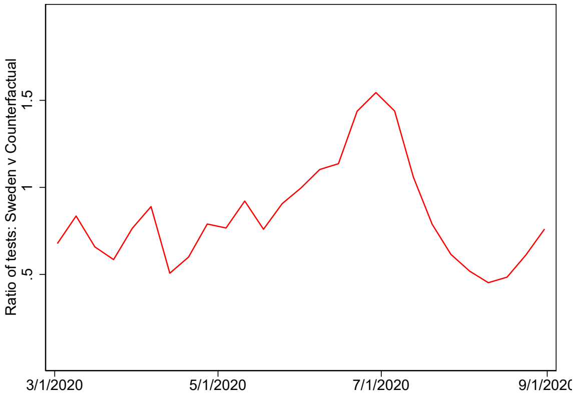


S2 Fig: Ratio of Tests. Weekly ratio of tests: Sweden vs the control unit (counterfactual) for baseline. Data source: European Centre for Disease Prevention and Control, 2020.Dong et al., 2020.

**S4. Correlation Plots.** Figure A.3 shows the correlation plot between the weights of the countries in the control unit and the timing of day 1 relative to Sweden. We find no systematic pattern in these data. For Specification A shown in the left panel the correlation is $-0.05$, while for Specification B it is 0.01.

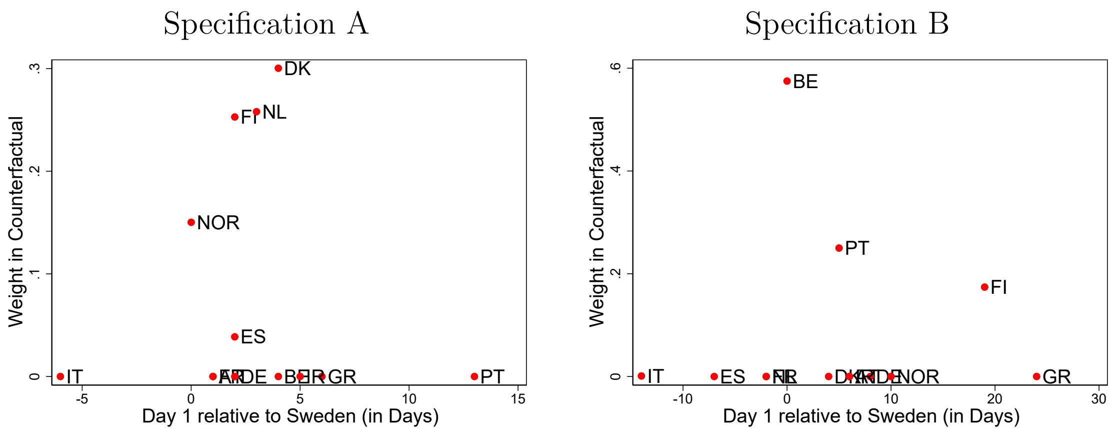


S3 Fig: Weight in control unit vs day one. Weights in control unit (vertical axis) vs difference from day 1 in a given country from day 1 in Sweden, in days (horizontal axis).

**S5. Counterfactual Scenarios Dropping Important Donor Countries.** Specification A: baseline matching infections. Specification A1-A4: alternative control units, for which each of the countries with a weight larger than 5% weight in baseline control unit, in turn, is excluded from donor pool. Specification B: baseline matching deaths. Specification B1-B3: alternative control units, for which each of the countries with a weight larger than 5% weight in baseline control unit, in turn, is excluded from donor pool.

S2 Table: Country weights.

| Specification | **A (Infections)** | A1 | A2 | A3 | A4 | **B (Deaths)** | B1 | B2 | B3 |
|---|---|---|---|---|---|---|---|---|---|
| Austria | **00.0** | 00.0 | 34.6 | 00.0 | 03.5 | **00.0** | 00.0 | 00.0 | 17.8 |
| Belgium | **00.0** | 00.0 | 00.0 | 12.7 | 00.0 | **57.5** | NaN | 52.6 | 58.0 |
| Denmark | **30.0** | NaN | 27.3 | 32.2 | 32.4 | **00.0** | 49.0 | 21.6 | 00.0 |
| Finland | **25.3** | 18.5 | NaN | 24.8 | 27.3 | **17.4** | 00.0 | NaN | 18.7 |
| France | **00.0** | 00.0 | 00.0 | 00.0 | 00.0 | **00.0** | 00.0 | 00.0 | 00.0 |
| Germany | **00.0** | 00.0 | 04.1 | 00.0 | 00.2 | **00.0** | 00.0 | 00.0 | 00.0 |
| Greece | **00.0** | 00.0 | 00.0 | 00.0 | 00.0 | **00.0** | 00.0 | 00.0 | 00.0 |
| Ireland | **00.0** | 00.0 | 00.0 | 00.0 | 00.0 | **00.0** | 00.0 | 00.0 | 00.0 |
| Italy | **00.0** | 00.0 | 00.0 | 00.0 | 00.0 | **00.1** | 00.0 | 00.5 | 00.0 |
| Netherlands | **25.8** | 34.0 | 02.9 | NaN | 36.5 | **00.0** | 36.5 | 00.0 | 05.4 |
| Norway | **15.0** | 32.4 | 31.1 | 20.9 | NaN | **00.0** | 00.0 | 00.0 | 00.0 |
| Portugal | **00.0** | 15.2 | 00.0 | 00.0 | 00.0 | **25.0** | 14.5 | 25.2 | NaN |
| Spain | **03.9** | 00.0 | 00.0 | 09.4 | 00.0 | **00.0** | 00.0 | 00.0 | 00.0 |

Table gives the weights in percent for each country in the optimized control unit for each specification (A1–A4 and B1–B3), where A and B are the baselines for infections and deaths, respectively. If table entry is *NaN*, country is excluded from the donor pool for the respective specification.

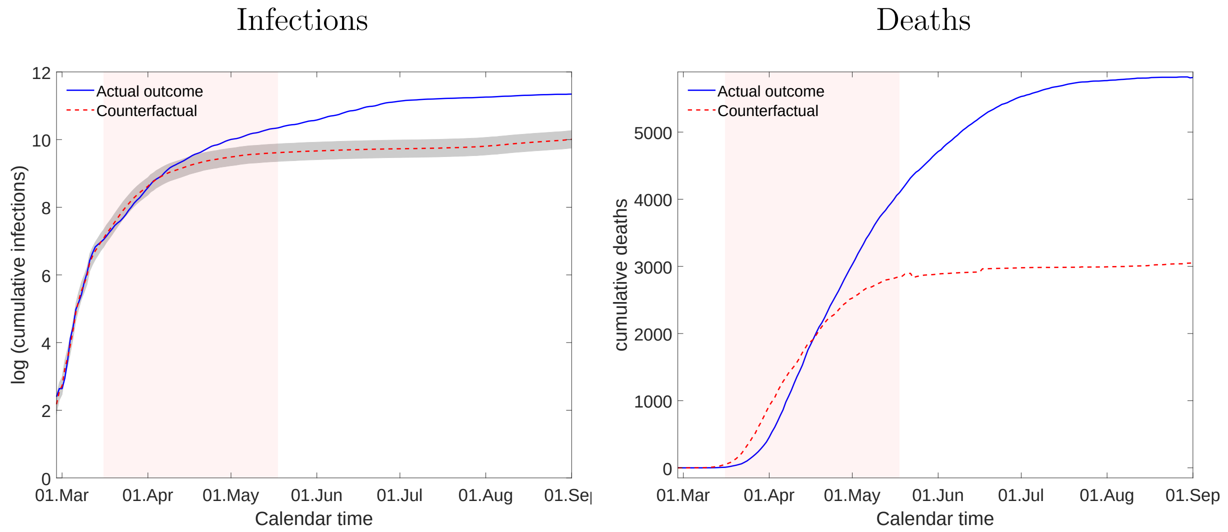


S4 Fig: Specification A. Specification A (Baseline Infections). Left panel shows infections, actual outcome (blue solid line) v counterfactual (red dashed line) in logs. Right panel shows deaths, actual outcome (blue solid line) v counterfactual (red dashed line). Counterfactual approximated by outcome of control unit, see Table A.2 for country weights of specification A (baseline). Gray shaded area: two standard deviations of difference between infections in Sweden and control unit during the first 13 d ays. P ink s haded a rea: l ockdown period.

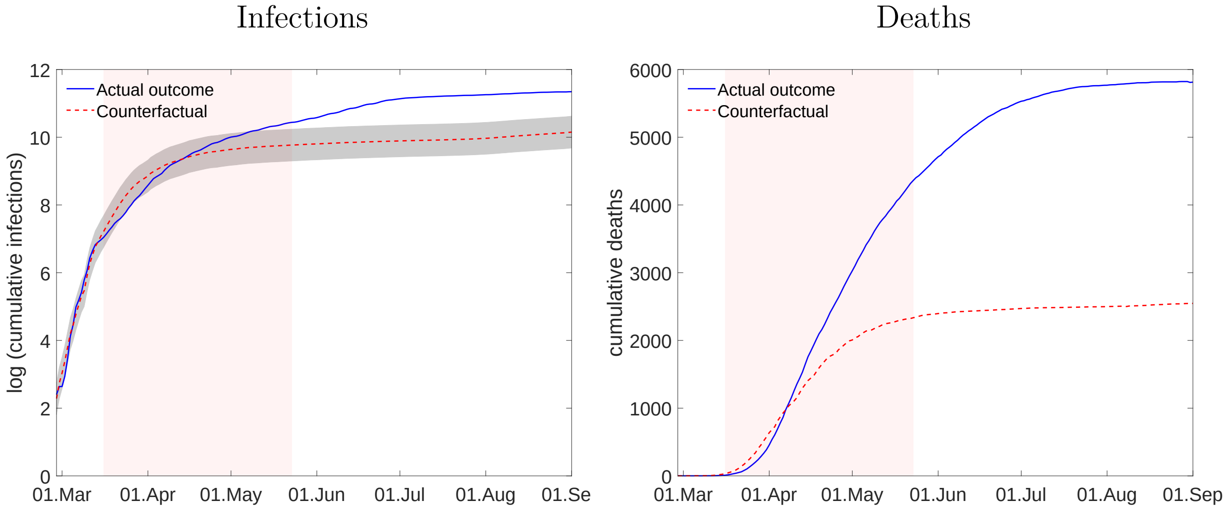


**S5 Fig**: Specification A1. Specification A1 (w/o Denmark). Left panel shows infections, actual outcome (blue solid line) v counterfactual (red dashed line) in logs. Right panel shows deaths, actual outcome (blue solid line) v counterfactual (red dashed line). Counterfactual approximated by outcome of control unit, see Table A.2 for country weights of specification A1. Gray shaded area: two standard deviations of difference between infections in Sweden and control unit during the first 13 days. Pink shaded area: lockdown period.

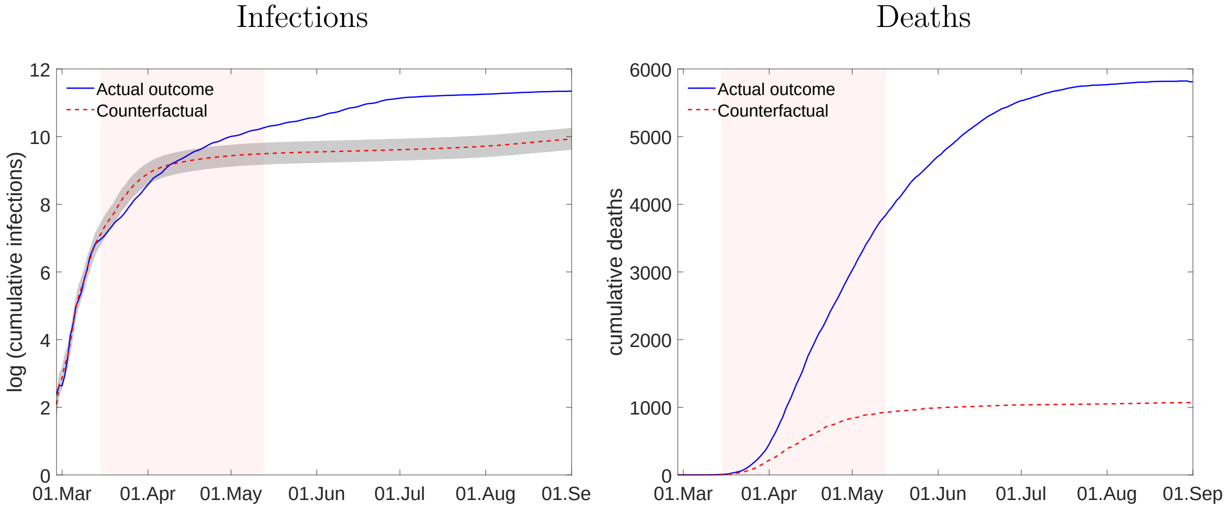


**S6 Fig**: Specification A2. Specification A2 (w/o Finland). Left panel shows infections, actual outcome (blue solid line) v counterfactual (red dashed line) in logs. Right panel shows deaths, actual outcome (blue solid line) v counterfactual (red dashed line). Counterfactual approximated by outcome of control unit, see Table A.2 for country weights of specification A2. Gray shaded area: two standard deviations of difference between infections in Sweden and control unit during the first 13 days. Pink shaded area: lockdown period.

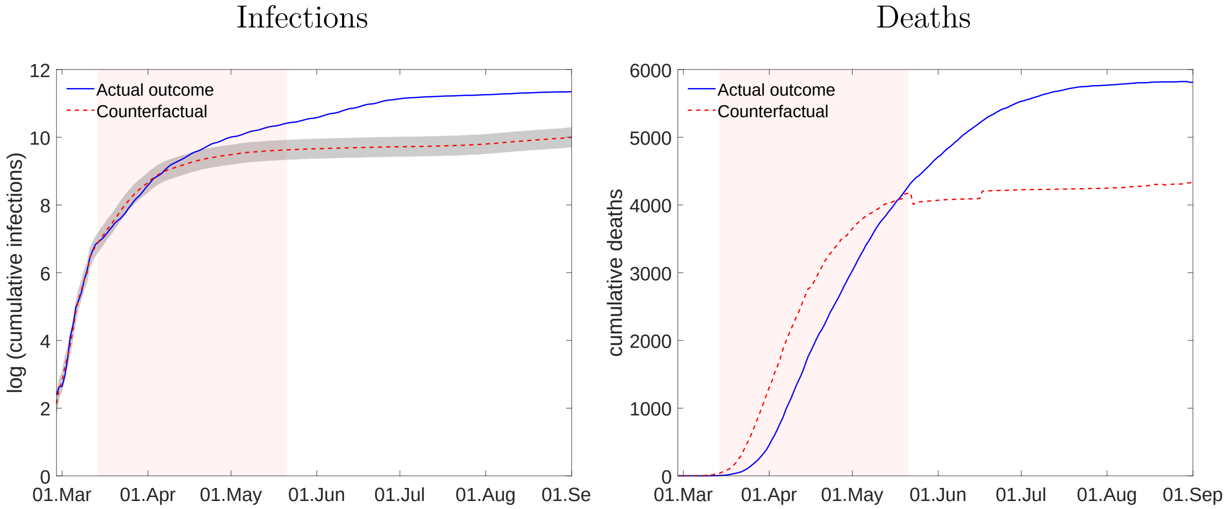


S7 Fig: Specification A3. Specification A3 (w/o Netherlands). Left panel shows infections, actual outcome (blue solid line) v counterfactual (red dashed line) in logs. Right panel shows deaths, actual outcome (blue solid line) v counterfactual (red dashed line). Counterfactual approximated by outcome of control unit, see Table A.2 for country weights of specification A3. Gray shaded area: two standard deviations of difference between infections in Sweden and control unit during the first 13 days. Pink shaded area: lockdown period.

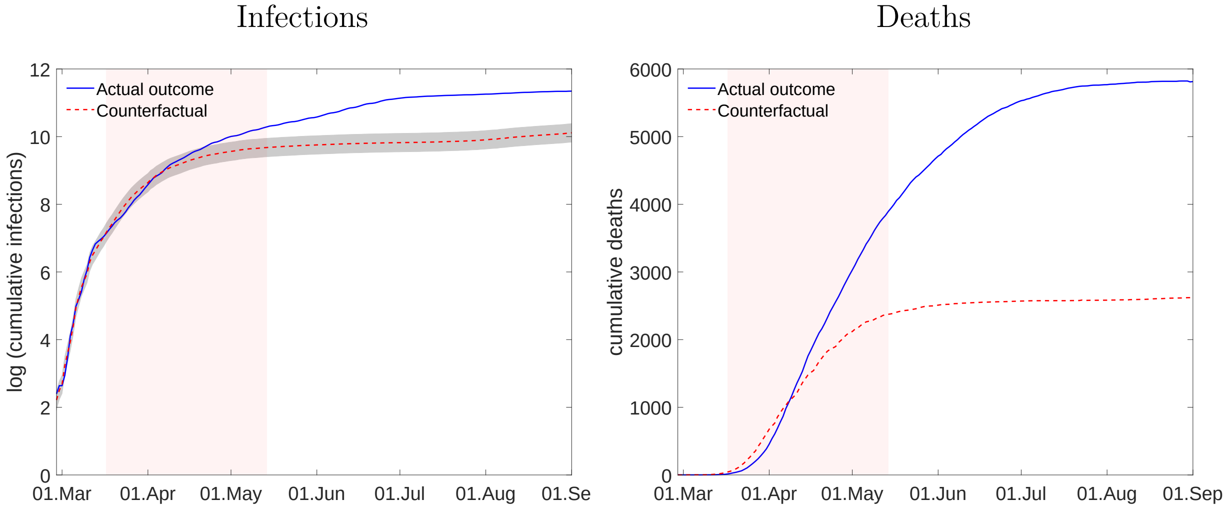


S8 Fig: Specification A4. Specification A4 (w/o Norway). Left panel shows infections, actual outcome (blue solid line) v counterfactual (red dashed line) in logs. Right panel shows deaths, actual outcome (blue solid line) v counterfactual (red dashed line). Counterfactual approximated by outcome of control unit, see Table A.2 for country weights of specification A4. Gray shaded area: two standard deviations of difference between infections in Sweden and control unit during the first 13 days. Pink shaded area: lockdown period.

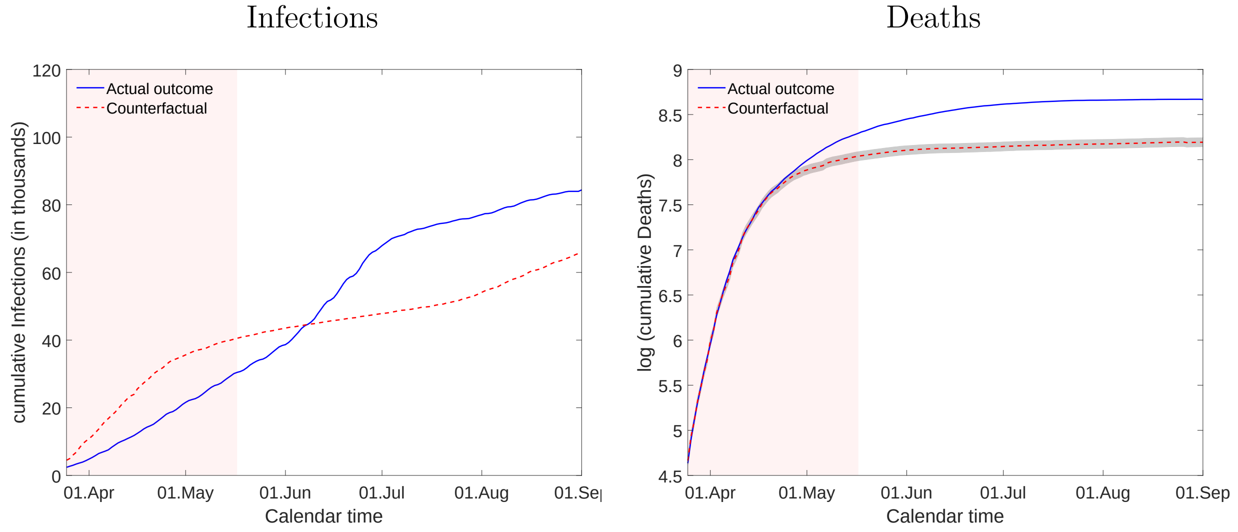


**S9 Fig**: Specification B. Specification B (Baseline Deaths). Left panel shows infections, actual outcome (blue solid line) v counterfactual (red dashed line) in logs. Right panel shows deaths, actual outcome (blue solid line) v counterfactual (red dashed line). Counterfactual approximated by outcome of control unit, see Table A.2 for country weights of specification B. Gray shaded area: two standard deviations of difference between infections in Sweden and control unit during the first 13 days. Pink shaded area: lockdown period.

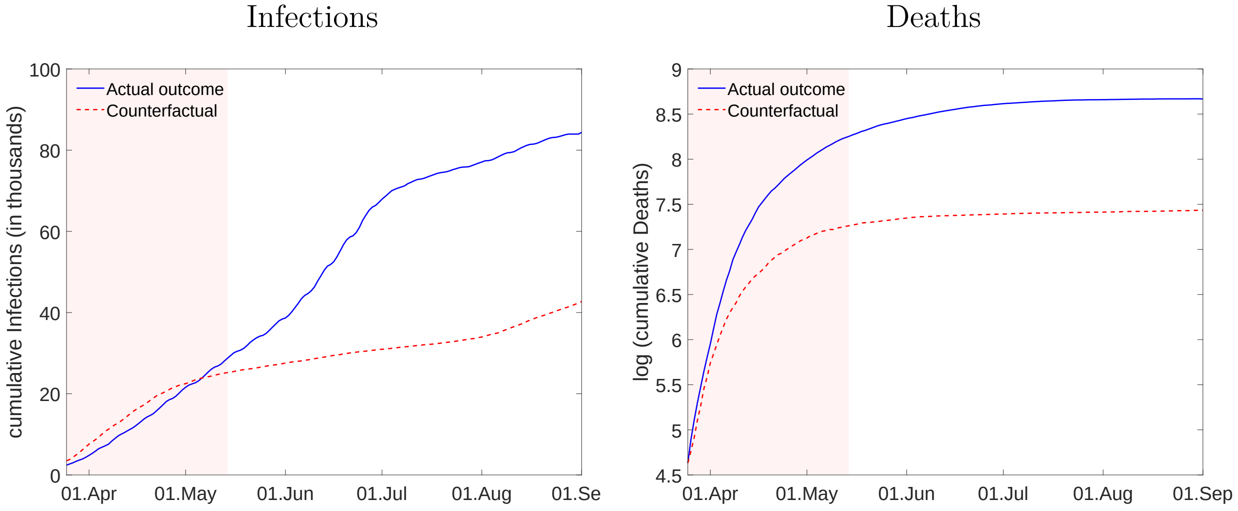


**S10 Fig**: Specification B1. Specification B1 (w/o Belgium). Left panel gives infections, actual outcome (blue solid line) v counterfactual (red dashed line) in logs. Right panel shows deaths, actual outcome (blue solid line) v counterfactual (red dashed line). Counterfactual approximated by outcome of control unit, see Table A.2 for country weights of specification B1. Gray shaded area: two standard deviations of difference between infections in Sweden and control unit during the first 13 days. Pink shaded area: lockdown period.

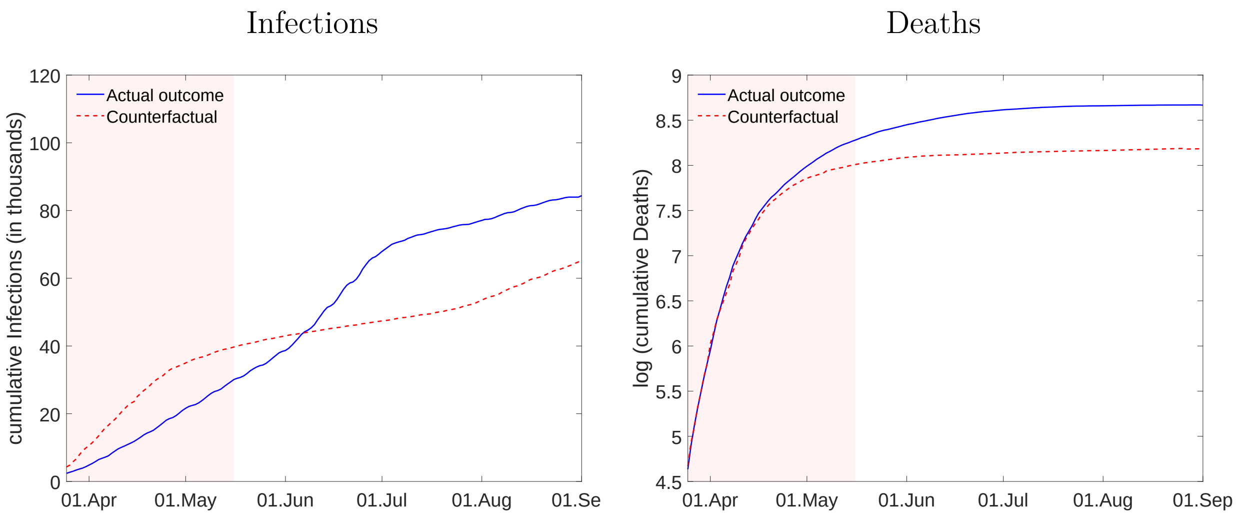


**S11 Fig:** Specification B2. Specification B2 (w/o Finland). Left panel shows infections, actual outcome (blue solid line) v counterfactual (red dashed line) in logs. Right panel shows deaths, actual outcome (blue solid line) v counterfactual (red dashed line). Counterfactual approximated by outcome of control unit, see Table A.2 for country weights of specification B2. Pink shaded area: lockdown period.

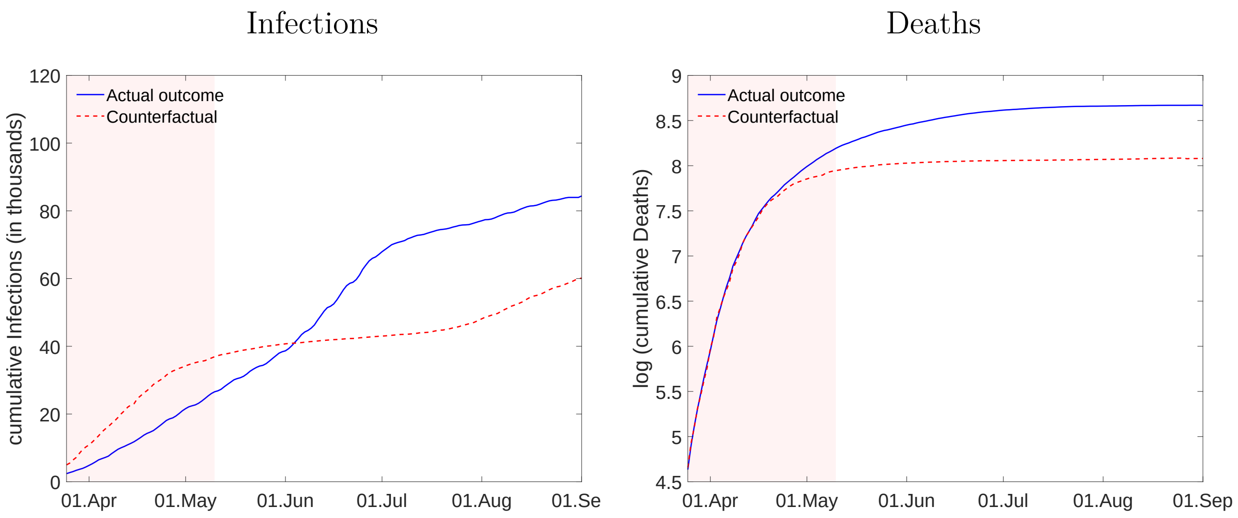


**S12 Fig:** Specification B3. Specification B3 (w/o Portugal). Left panel shows infections, actual outcome (blue solid line) v counterfactual (red dashed line) in logs. Right panel shows deaths, actual outcome (blue solid line) v counterfactual (red dashed line). Counterfactual approximated by outcome of control unit, see Table A.2 for country weights of specification B3. Pink shaded area: lockdown period.

**S6. Additional Robustness.** We do some additional robustness specifications based on the timing of Day 1, both for the baseline matching Infections (A) and Deaths (B).

S3 Table: The lockdown effect on COVID-19 infections and deaths – different timings

| | | Infections | | Deaths | |
|---|---|---|---|---|---|
| | | Actual | Counterfactual | Actual | Counterfactual |
| **A)** | **Baseline (Matching Infections)** | **83,458** | **21,150** | **5,806** | **3,013** |
| | (1 infected per 1,000,000) | | **-75%** | | **-48%** |
| AI) | 1 infected per 5.000,000 | 83,256 | 20,461 | 5,799 | 2,274 |
| | | | -75% | | -61% |
| AII) | 1 infected per 100,000 | 83458 | 31432 | 5806 | 4721 |
| | | | -62% | | -19% |
| **B)** | **Baseline (Matching Deaths)** | **79,122** | **57,655** | **5,795** | **3,573** |
| | (1 dead per 100,000) | | **-27%** | | **-38%** |
| BI) | 1 dead per 1,000,000 | 82,051 | 56,940 | 5,809 | 3,148 |
| | | | -31% | | -46% |
| BII) | 1 dead per 50,000 | 77,053 | 53,517 | 5,767 | 3,643 |
| | | | -31% | | -37% |

Notes: New infections and deaths since start of lockdown up until September 1, 2020. Figures for the actual outcome differ across specifications because the start of the lockdown period varies with the composition of the control unit. Panel A: baseline matching infections (see Figure **??**). Panel B: baseline matching infections (see Figure **??**). AI-AII and BI-BII: alternative control units, for which the definition of Day 1 is varied.

**S7. Placebo Excercise.** We conduct placebo excercises for both specification A and B. That is, for each country of the donor pool, we construct a counterfactual – similar to the one for Sweden in the baseline – from the remaining donor pool. We then compare the relative deviation of each country from its counterfactual before and after the end of the matching period Born et al., 2019. Formally, we define the relative root mean squared prediction error ($RMSE$) as:

$$RMSE = \frac{RMSE_{Post}}{RMSE_{Pre}} = \frac{\sqrt{\frac{1}{T-T_0-1}\sum_{t=T_0}^{T}\left(x_{1,t} - x_{0,t}w - (x_{1,T_0} - x_{0,T_0}w)\right)^2}}{\sqrt{\frac{1}{T_0-1}\sum_{t=1}^{T_0-1}\left(x_{1,t} - x_{0,t}w\right)\right)^2}} \quad (1)$$

where $w$ denotes the vector of country weights, $x_{1,t}$ is the outcome in Sweden in $t$ and $x_{0,t}$ is the vector of respective outcomes in the donor pool in $t$. $T_0 = 13$ gives the number of periods used for matching the counterfactual and $T$ is the overall number of observations. Here, we compute statistics until September 1. Note that normalise the post-treatment prediction error to zero at the treatment date.

S4 Table: Relative RMSE.

| Country | Spec. A (Infections) | Spec. B (Deaths) |
|---|---|---|
| **Sweden** | **7.36** | **13.43** |
| | | |
| Austria | 3.34 | 5.39 |
| Belgium | 1.71 | 2.03 |
| Denmark | 0.30 | 0.44 |
| Finland | 1.92 | 0.36 |
| France | 1.10 | 1.38 |
| Germany | 0.85 | 1.02 |
| Greece | 3.98 | 0.82 |
| Ireland | 3.40 | 2.28 |
| Italy | 0.58 | 5.14 |
| Netherlands | 0.22 | 4.53 |
| Norway | 3.60 | 2.19 |
| Portugal | 4.74 | 7.43 |
| Spain | 0.61 | 0.32 |

Table shows the RMSE between the matching period and the time after the matching period until September 1.

# References

Born, Benjamin, Gernot Müller, Moritz Schularick, and Petr Sedláček (2019). "The costs of economic nationalism: evidence from the Brexit experiment". *The Economic Journal 129* (623), 2722–2744.

Dong, Ensheng, Hongru Du, and Lauren Gardner (2020). "An interactive web-based dashboard to track COVID-19 in real time". *Lancet Infect Dis 20* (5), 533–534.

European Centre for Disease Prevention and Control (2020). *Data on testing for covid-19 by week and country.* `https://www.ecdc.europa.eu/en/publications-data/covid-19-testing`.

Google (2020). *Google COVID-19 Community Mobility Reports.* `https://www.google.com/covid19/mobility/`. Accessed: 01.07.2020.

OECD (2020). *Quarterly gdp (indicator). (accessed on 17 september 2020).* `https://doi.org/10.1787/b86d1fc8-en`.

Worldbank (2019 Revision[a]). *Population ages 65 and above (% of total population), sp.pop.65up.to.zs.*

——— (2019 Revision[b]). *Population, total, sp.pop.totl.*

——— (2019 Revision[c]). *Urban population (% of total population), sp.urb.totl.in.zs.*
